# Children Intoxicated with Psychoactive Substances: The Health Status on Admission to Hospital Based on Medical Records

**DOI:** 10.3390/jcm13061771

**Published:** 2024-03-20

**Authors:** Dorota Kosiorek, Jolanta Lewko, Ewa Romankiewicz

**Affiliations:** 1 Department of Infectious Diseases, Provincial Specialist Children’s Hospital in Olsztyn, 10-561 Olsztyn, Poland; dorakosi@wp.pl (D.K.); e.romankiewicz@wssd.olsztyn.pl (E.R.); 2 Department of Primary Health Care, Medical University of Bialystok, 15-089 Bialystok, Poland

**Keywords:** children, psychoactive substances, pain, Poisoning Severity Score (PSS)

## Abstract

**Introduction:** Alcohol is the most common psychoactive substance among young people. The use of psychoactive substances gives rise to a number of health, social, moral and economic problems. The aim of the study was to characterise the symptoms reported by children and adolescents presenting with psychoactive substance intoxication on admission to hospital. **Methods:** The study included a group of 303 patients aged 0 to 18 years, diagnosed with psychoactive substance intoxication. This retrospective study assessed medical records of patients hospitalised at the Provincial Specialist Children’s Hospital in Olsztyn between 2016 and 2018. **Results:** Body temperature on admission varied depending on the type of psychoactive substance taken (χ^2^ = 14.12, *p* < 0.006). Girls were more likely to have an increased heart rate than boys. An analysis of the symptoms of intoxication over the years 2016–2018 showed significant differences in the incidence of the following symptoms: disturbed consciousness (χ^2^ = 8.75, *p* < 0.01), visual disorders (χ^2^ = 8.02, *p* < 0.02), loss of consciousness (χ^2^ = 37.71, *p* < 0.000001), drowsiness (χ^2^ = 7.33, *p* < 0.03), hypothermia (χ^2^ = 18.95, *p* < 0.00008) and gait disturbances (χ^2^ = 13.08, *p* < 0.002). **Conclusions:** Girls are more likely to use stimulants such as alcohol and cigarettes, while boys are more likely to opt for designer drugs. The number of patients hospitalised more than once increases every year. Gastrointestinal intoxication with psychoactive substances was most common. The most severe intoxication was associated with alcohol intake, while the most intense pain was reported by patients poisoned with other psychoactive substances.

## 1. Introduction

In addition to their mood-elevating and hallucinogenic effects, psychoactive substances, including designer drugs, cause hyperactivity and psychomotor arousal and may lead to dependence syndrome if used regularly. However, it should be noted that the use of psychoactive substances also gives rise to a number of health, social, moral and economic problems [1].

The European School Survey Project on Alcohol and Drugs (ESPAD), a nationwide research survey on the use of psychoactive substances among school youth, has so far shown that alcohol is the most common psychoactive substance among teenagers. About 83.8% of students from the younger group and 95.8% of students from the older group consumed alcohol at least once in their lifetime, most of whom admitted to being above the threshold of intoxication. The report showed that crossing the threshold of intoxication after drinking alcohol was experienced at least once in their lives by one third of fifteen–sixteen-year-olds (33.3%) and over half of students from the older group (56.6%), while in the last 30 days, 11.3% of third-grade middle school students and 18.8% of second-grade high school students became very drunk. The results of current nationwide research have shown that drinking alcohol among Polish youth has become an almost daily norm. Hospitalisations after alcohol poisoning still constitute a large percentage of all hospitalised patients in hospital emergency departments and paediatric departments.

Nicotine is the second most widely distributed legal psychoactive substance used by school children. However, the use of illegal substances is becoming an increasing problem, with 25% of younger and 43% of older students declaring having used marijuana or hash at least once in their lifetime. Polish 15- and 16-year-olds rank first in Europe in terms of using sedatives and sleep aids without a doctor’s recommendation. The lack of attractive leisure activities promotes emotional disorders among young people, triggering negative moods and depression and anxiety disorders, which may in turn promote the use of alcohol and other psychoactive substances [2,3,4].

The aim of the study was to characterise the symptoms reported by children and adolescents intoxicated with psychoactive substances on admission to hospital.

## 2. Materials and Methods

### 2.1. Study Group

The study included a group of 303 patients aged 0 to 18 years, diagnosed with psychoactive substance poisoning. This retrospective study assessed medical records of patients hospitalised in the Provincial Specialist Children’s Hospital in Olsztyn from 1 January 2016 to 31 December 2018 due to intoxication with psychoactive substances. 

### 2.2. Study Design

On medical interview, the dose of the ingested psychoactive substance was estimated, the level of consciousness was assessed using the Glasgow Coma Scale (GCS), the severity of intoxication was assessed with the Poisoning Severity Score (PSS) and pain was assessed using 3 Pain Rating Scales in different age groups. Additionally, self-mutilation, injuries, dehydration, heart rate, blood pressure, respiration, oxygen saturation and temperature were assessed, as well as BMI which was calculated during physical examination on admission.

### 2.3. Measures

The Paediatric Glasgow Coma Scale (pGCS) is used to assess the extent of impaired consciousness in children <3 years of age. The score range is from 3 to 15, with 3 for completely unresponsive (worst prognosis) and 15 for responsive. Mild (13–15), moderate (9–12) and severe consciousness disorders (6–8) can be distinguished [5]. 

Poisoning Severity Score (PSS) is an international classification of poisoning severity for both adults and children. It can be used in the case of any acute poisoning regardless of the type and number of agents involved. The PSS should take into account the overall clinical course and be applied according to the most severe symptomatology (including both subjective symptoms and objective signs). It can be completed at any time during hospitalisation, provided that the time of data collection is precisely defined. This scale does not include risks and threats based on data, such as the amount of substance consumed or xenobiotic blood levels [6]. 

Faces Pain Scale (FACES) uses a series of 6 facial expression illustrations to outline a scale of pain severity. The patient is asked to point to a face that shows how much pain they feel at the time of the examination. Each face is assigned a numeric value from 0 to 10, where 0 represents “no pain” and 10 represents “worst possible pain”. The higher the score, the more severe the pain. This scale is used in children >3 years of age and in adults with difficulties with verbal communication. This scale is recommended by the International Association for the Study of Pain (IASP) [7].

Numerical Rating Scale (NRS) is an 11-point numeric scale (10 cm) ranging from 0, representing “no pain”, to 10 for the most severe pain. It is most often used in children >7 years of age with logical verbal contact [8]. 

The Face, Legs, Activity Cry and Consolability (FLACC) is a tool to assess 5 behaviours corresponding to the sensation of pain in children (facial expression, leg position, general activity, crying and effort needed to console the patient). Each category is scored from 0 to 2 and then the partial scores are summed to give a total score from 0 (no pain/full comfort) to 10 (severe pain/discomfort). A score of ≥3 means the need for analgesics and a pain monitoring card. The tool is used in patients aged 0–3 years and children without logical verbal contact (including intubated patients and patients with severe intellectual disabilities) [9].

### 2.4. Procedure and Ethical Considerations

The study was approved by the Bioethics Committee of the Medical University of Białystok (Resolution No. R-I-002/399/2018).

### 2.5. Statistical Analysis

Statistical analysis was performed using Microsoft Excel 2019 and TIBCO Statistica 3.3PL. The Kruskal–Wallis Test was used to assess the homogeneity of the distribution of variables in a given population. The chi-square (χ^2^) test was used to assess the relationship between two qualitative variables. Results with a significance coefficient of *p* ≤ 0.05 were considered statistically significant. 

## 3. Results

Medical records of 303 paediatric patients with confirmed psychoactive substance intoxication were analysed. In the years 2016–2018, a total of 66,035 patients were hospitalised in the Provincial Specialist Children’s Hospital in Olsztyn, including 303 cases of intoxication with psychoactive substances, which accounts for 0.45% of all hospitalised patients. The mean age of hospitalised patients was 14.83 ± 2.88 with a median of 15.5 years. Girls and boys accounted for 158 (52.15%) and 145 (47.85%) hospitalised cases, respectively. There were no hospitalisations of patients in the 5–8 age group. The details are presented in Table 1.

The clinical symptoms observed in patients intoxicated with psychoactive substances varied. The patients were assessed for consciousness using the GCS on admission. Mild (GCS 13–15), moderate (GCS 9–12) and severe (GCS 6–8) consciousness disorders were found in 78.55%, 18.15% and 3.30% of patients, respectively. In case of severe GCS, data were collected from parents or guardians. 

The severity of intoxication was assessed with PSS. The majority of patients with psychoactive substance intoxication assessed with PSS presented with mild symptoms (58.75%), while 23.1% of patients had no symptoms of poisoning. Moderate and severe poisoning was found in 14.19% and 3.96% of patients, respectively.

No injuries or cases of self-harm were found in 72.94% of patients with symptoms of psychoactive substance intoxication. Injuries or self-harm occurred in 27.06% of patients.

In the case of patients with injuries (*n* = 82), self-harm accounted for 53.66% and one third of patients (30.49%) suffered from abrasions. Head injuries accounted for 15.85% of all injuries.

Each patient was assessed for pain on admission to the Department of Emergency. In most of the analysed cases, 84.49% of patients did not report pain on admission. Mild pain occurred in 5.94% of cases. Moderate and severe pain was reported by the same number of patients (3.96%), while very severe pain was observed in 1.65% of patients. 

In most cases (90.46%), no signs of dehydration were observed. Dehydration occurred in 9.24% of patients. 

More than half of the patients (64.69%) had a normal body temperature on admission. Approximately one in four patients (24.42%) had a subnormal temperature, whereas a subfebrile condition was found in 10.89% of the study group.

The temperature distribution on admission to hospital was similar in male and female groups (χ^2^ = 2.47, *p* < 0.29) and in different age groups (χ^2^ = 13.47, *p* < 0.19). Body temperature on admission varied depending on the type of psychoactive substance taken (χ^2^ = 14.12, *p* < 0.006). A subnormal temperature occurred significantly more often in patients after alcohol intake (33.59%). A low-grade fever was more common in patients after taking designer drugs (13.7%).

Body temperature distribution on admission differed significantly depending on the alcohol concentration (χ^2^ = 28.94, *p* < 0.001). A subnormal temperature was significantly more common in patients after consuming alcohol at >2–2.5‰. A low-grade fever occurred significantly more often in patients after consuming alcohol at <1.5‰. The details are presented in Table 2.

More than half of the study group (55.78%) had normal blood pressure (BP) on admission to the Department of Emergency. Approximately one in three patients (29.37%) had elevated BP, while 14.85% of patients presented with low BP. The distribution of BP on admission was similar in male and female groups and was not statistically significant (χ^2^ = 0.47, *p* < 0.78). The distribution of blood pressure on admission did not differ statistically significantly across age groups (χ^2^ = 13.28, *p* < 0.20).

More than half (65.35%) of patients in the study group had an increased heart rate (HR), one in three patients (32.34%) had a normal HR, whereas a reduced HR was observed in 2.31% of the study group.

HR values did not differ significantly statistically (χ^2^ = 5.32, *p* < 0.86) in the age groups, whereas there were significant differences in the distribution of the HR values by gender (χ^2^ = 6.21, *p* < 0.04). Girls were significantly more likely to have an increased HR than boys. The details are presented in Table 3.

The majority of patients (93.40%) in the study group had normal blood saturation. Abnormal saturation <95% was found in 6.6% of patients.

A normal breathing rate on admission was observed in 71.62% of patients. One in four patients (24.75%) presented with an increased breathing rate, whereas a reduced breathing rate was found in 3.63% of patients. The distribution of the breathing rate by gender (χ^2^ = 0.77, *p* < 0.88) and age (χ^2^ = 6.07, *p* < 0.70) was similar; the differences between the groups were not statistically significant.

The BMI analysis showed that the majority of patients (81.85%) had a normal body weight. A body weight deficiency occurred in 4.29% of patients, whereas overweight and obese patients accounted for 5.94% and 7.92%, respectively.

Alcohol (28.38%) and cigarettes (27.39%) were the most frequently used stimulants in the study group of children and adolescents, while drugs and designer drugs were used by 8.91% and 7.92% of patients, respectively. Approximately one in three patients (31.02%) declared no use of stimulants. Medical history data on the stimulants used were missing in 20.46% of patients.

In the group of patients who declared the use of stimulants, alcohol consumption was declared by 55.81% of girls and 44.19% of boys. Furthermore, 59.04% of girls and 40.96% of boys admitted that they smoked cigarettes. In this group, girls were more likely to use stimulants such as alcohol and cigarettes. However, boys were more likely to opt for designer drugs (Table 4).

Adolescents between 15 and 18 years of age declared the use of the greatest amount of stimulants. The details are presented in Table 5.

The results of the analysis of the frequency of poisonings with a given group of psychoactive substances in particular age groups showed that the most common cases were poisonings with psychoactive substances—48.18% of all poisonings. Patients aged 13–16 were significantly more likely to be hospitalised due to alcohol poisoning. However, patients aged 17–18 were hospitalised significantly more often due to mixed poisonings. The distribution of the number of patients in particular age groups depending on the type of poisoning was significantly different (χ^2^ = 42.74, *p* < 0.00001). 

The age difference in the cohort of participants varied from 0 to 18 years and a difference in psychoactive substance poisoning was observed between a newborn who could have been poisoned in utero and 17-year-olds who voluntarily took a psychoactive substance.

In the group of children aged 0 to 4 years, all poisonings with psychoactive substances were accidental. Poisonings occurred both at home and outside. Children in the groups who were 0–4 and 9–10 years old were poisoned with psychoactive substances, most of them being medications.

An analysis of the reasons for reporting to hospital found that suspected alcohol intoxication accounted for almost half of the cases (42.24%). This was followed by a loss of consciousness (15.51%), their state after taking a psychoactive substance (13.53%), the intentional consumption of medications (12.54%), taking medications (11.22%) and behavioural disorders (10.23%). The intake of designer drugs was another reason for admission (6.6%).

Patients who experienced vomiting/nausea and dizziness accounted for 5.94% and 2.97% of cases, respectively. Suicide attempts were reported for 1.98%, while suspicion of rape and loss of consciousness were reported for 1.32% of patients. Visual disturbances (0.99%), chest pain (0.33%) and the intake of fluid of unknown origin (0.33%) accounted for less than 1%. 

First-time hospitalisations due to psychoactive substance intoxication accounted for 80.53%. Nearly one in ten patients (11.22%) were hospitalised for the second time and 8.25% of patients were hospitalised for the third or fourth time.

The analysis of the distribution of the number of first-time hospitalisations showed a decrease from year to year (χ^2^ = 14.58, *p* < 0.006), while the number of patients hospitalised for the second, third or more times has increased. A gastric lavage was performed in 3.16% of hospitalised patients in 2016 and in 10.68% of children in 2018.

An analysis of cases of alcohol intoxication by the month in which the poisoning occurred found that the largest number of cases occurred in the following months: June (8.91%), August (5.94%) and November (5.94%). There were no statistically significant differences in the incidence of alcohol intoxication in individual months over subsequent years.

The incidence rates of intoxication with other psychoactive substances in each month ranged from 1.98% in July to 6.93% in March. The highest number of poisonings with psychoactive substances occurred in March (6.93%), June (6.60%) and August (6.60%). 

Most poisoning events occurred by ingestion (83.50%, *n* = 253), followed by inhalation (15.84%, *n* = 48). An intravenous route was reported for one child with poisoning symptoms. There were no statistically significant differences in the distribution of routes of intoxication over the analysed years (χ^2^ = 4.84, *p* < 0.56). The details are shown in Table 6.

All children who ingested a toxic substance developed clinical manifestations of poisoning (*n* = 303). The simultaneous occurrence of more than one symptom was the most common finding in the analysed group of intoxicated patients. An analysis of the poisoning symptoms in the years 2016–2018 found significant differences in the number of cases for the following symptoms: impaired consciousness (χ^2^ = 8.75, *p* < 0.01), impaired vision (χ^2^ = 8.02, *p* < 0.02), loss of consciousness (χ^2^ = 37.71, *p* < 0.000001), drowsiness (χ^2^ = 7.33, *p* < 0.03), hypothermia (χ^2^ = 18.95, *p* < 0.00008) and gait disturbance (χ^2^ = 13.08, *p* < 0.002). The results of the analysis of the prevalence of clinical symptoms in the analysed period are shown in Table 7.

The majority of patients (78.55%) with symptoms of psychoactive substance poisoning were conscious at the time of admission. About one in five patients (18.15%) were unconscious. Intubation was needed in 3.3% of patients. The distribution of the consciousness/mental states of intoxicated patients was similar in the analysed period (χ^2^ = 3.17, *p* < 0.52). The details are shown in Table 8.

The Kruskal–Wallis H test was used to compare the GCS scores, the severity of intoxication and pain on admission due to the lack of parametric distribution in these scales. The significance level was set at *p* < 0.05.

When comparing the GCS scores across the groups of men and women, similar results were obtained, whereas by the type of intoxication, the highest severity of poisoning was recorded for alcohol consumption and the same severity was observed for intoxication with other psychoactive substances and mixed substances. The results of the analysis are shown in Table 9.

The analysis showed a comparable severity of intoxication by gender. In contrast, the most severe intoxication was noted after alcohol consumption, which was comparable to that of mixed substances. The severity of intoxication was shown to be significantly correlated with the type of intoxication (*p* = 0.002). The details are shown in Table 10.

The severity of pain on admission was comparable in the groups by gender. The lowest intensity was reported by those who had consumed alcohol, while the most severe pain was reported by patients intoxicated with other psychoactive substances. The details are presented in Table 11.

## 4. Discussion

The study showed that the vast majority of patients intoxicated with psychoactive substances (91.09%) were transferred to the Hospital Emergency Department by an Emergency Medical Team (EMT), indicating a sudden onset of poisoning, as well as the need for prompt and professional medical assistance. The vast majority of patients (96.7%) were admitted without a referral, while only a small group accounting for 3.3% were admitted with a referral. EMTs play an important role at this point as according to our research, only 8.25% of patients were brought to the hospital directly by their parents. It should be noted that the number of emergency admissions with the presence of police was significantly higher in subsequent years and that boys are more often admitted to the hospital assisted by the police than girls. Similar results were obtained in a nine-year analysis by Jackowska [10], where 57.2% of patients were transported to hospital by an EMT and 5.8% were referred by the night medical service. Drug and alcohol intoxication remain the main cause of emergency admissions in the study group of children, which corresponds with findings in other European countries, such as Spain, or non-European countries, such as Iran and Israel. However, in these countries, alcohol consumption has been a problem among male adolescents, while our study showed that drug and alcohol consumption is more common among girls [11,12,13,14,15,16].

Although little is known about new psychoactive substances (NPSs), some can lead to a number of serious adverse effects that can manifest with unconsciousness, epileptic seizures and other psychopathological disorders. Designer drugs are currently one of the most readily available substances that almost anyone can buy online. They also pose the highest health risk due to their unknown content. Our analysis has shown that male adolescents were more likely to use NPSs, as confirmed in both the Polish and global literature [17,18,19,20].

The study group of children was evaluated for venous blood alcohol levels. These levels were always measured with the same method and in the same laboratory in the Provincial Specialist Children’s Hospital in Olsztyn. Special attention should be paid to the blood alcohol levels in patients admitted to hospital, which ranged from 0.4‰ to 3.98‰. These observations are in line with studies by other authors, who also pointed to the problem of alcohol poisoning among children and adolescents. High blood alcohol levels are reported both in the Polish and world literature. Bouthoorn [21] described a maximum blood alcohol level of 5‰ among Danish children.

Based on medical records, children and adolescents were evaluated for the severity of poisoning using PSS and GCS. Mild, moderate and severe poisoning was found in more than half (58.75%), 14.19% and 3.96% of children, respectively. GCS showed mild consciousness disorders in 78.55% and a lack of consciousness in 3.30% of patients on admission. The frequent and excessive consumption of alcohol and other psychoactive substances can cause symptoms of multiorgan dysfunction. Imbalance and euphoria were observed with mild intoxication. Slurred speech, visual disturbances, drowsiness or impaired consciousness were observed in children with severe poisoning. The symptoms themselves, as well as their sequence and severity, depended primarily on individual susceptibility, the amount of substances taken, their metabolism and whether the patient used only one agent or several different substances at the same time. These symptoms were also observed by Panasiuk [22], who pointed out that the clinical picture of intoxicated children was highly variable. The most common symptoms mentioned by the author included agitation, delirium, headaches and dizziness, drowsiness, impaired consciousness, coma, nausea and vomiting. The use of psychoactive substances by children and adolescents often compromises self-control and may threaten both health and life (e.g., hypothermia in the winter, injuries or engaging in risky behaviours such as aggressive outbursts, accidents, rape, etc.). This problem was also pointed out by Graddy et al. [20] and Cook et al. [23]. 

The intoxicated patients presented with varied clinical symptoms on admission to the Hospital Emergency Department. It is very important to secure all biological materials at the time of admission, as well as, if possible, all psychoactive substances at the scene of the event for a correct diagnosis and the implementation of the appropriate treatment. The type of toxic substance and the time elapsed since its ingestion determine further treatment. Ambiguous yet frequently observed symptoms of intoxication with psychoactive substances include CNS and gastrointestinal manifestations. According to our data, the symptoms in patients admitted to the Department of Emergency after intoxication with psychoactive substances included disturbed consciousness, visual disturbances, loss of consciousness, drowsiness, gait and balance disturbances, as well as hypothermia. The fact that the patients who were unconscious on admission accounted for 18.15% of the total number of poisoned patients is alarming. All children admitted to the Emergency Department were put under hospital observation with the monitoring of basic vital parameters. In most cases, intravenous hydration was initiated and treatment was started depending on the clinical condition. The purpose of hydration is to achieve forced diuresis to eliminate toxins from the body. During hospitalisation, intravenous hydration in the form of Optilyte, Glucosol 2/1 and 0.9% NaCl was used. The authors recommend performing gastric lavage when no more than 1 h has elapsed since the ingestion of the poisonous substance or when the ingested substance has slowed gastrointestinal peristalsis, as well as when prolonged-release drugs have been taken. In the case of children and adolescents intoxicated with psychoactive substances who were admitted to the hospital without caregivers, determining the substance and the time of ingestion is extremely difficult, as also confirmed by other authors [14,24,25]. In the present study, the vast majority of patients received treatment during their stay in the ED, while no treatment was administered to only 15.18% of patients due to their good clinical condition, short stay in the ward or discharge on parental request before the initiation of the therapeutic process.

Vital functions were monitored in all children admitted due to psychoactive substance intoxication. Dehydration was found in 9.24% of hospitalised patients. A normal temperature in babies from 0 to 12 months of age is considered to be around 36.5–37.5 °C, the same as from 1 to 11 years old and above, for which the normal body temperature range is also 36.5–37.5 °C. A subnormal temperature, which was found in 24.42% of cases, was a disturbing symptom. The decrease in body temperature was related to hypothermia following alcohol consumption. 

The normal heart rate for children varies with age—children between 0 and 12 months of age have a heart rate of 70–190 bpm, kids over 1 year old to 11 years old have a heart rate of 52–156 beats per minute and teenagers above 12 years of age have an HR between 60 and 110 bpm [26]. 

Normal and high blood pressure depends on a child’s age, sex and height. For children who are 13 and older, the normal blood pressure is 120/80 mmHg or lower [27].

Abnormal BP (elevated or decreased) and HR parameters were found in 44.22% and 67.66% of patients, respectively. It is noteworthy that the vast majority of patients experienced no pain, which was related to the type of psychoactive substance taken. Severe and very severe pain was present in 5.61% of children. A total of 248 (81.85%) children were presented with mild intoxication or no symptoms of intoxication, while moderate or severe poisoning was found in 55 (18.15%) patients. After the applied treatment, the patients’ general condition improved relatively quickly and a hospital stay of more than three days was needed in only 14.85% of patients.

Health care providers should compare vital signs (heart rate, blood pressure, temperature and more) with current guidelines before they consider them appropriate for this age category. With measurements taken by different people, measurement errors can occur [28].

Recent studies have shown a decreasing trend in the use of NPSs. However, as shown by Burda [29], the situation in 2015 when 7284 medical interventions due to NPS use were reported, of which 1517 were among children under the age of 18 years, and the situation in 2016 with 4369 interventions, including 758 children and adolescents, indicate that despite the less common use of these substances among adolescents, the number of problems due to these poisonings may be increasing. A downward trend in poisonings was observed, with the rates in this group estimated at 43% in 2013, followed by 26.5% in 2015, 23.7% in 2016, 20.5% in 2017 and about 16% in 2018. However, in addition to NPSs, other psychoactive substances frequently used by adolescents, including alcohol or cigarettes, are available on the market. The effectiveness of the treatment process, which combines the stages of recovery and returning to full mental and physical fitness, is an important aspect of hospitalisation. The use of psychoactive substances has become a clinical problem, posing challenges for clinicians working not only in mental health units, but also in emergency and paediatric departments. Thus, early detection, monitoring and limiting the availability of psychoactive substances are areas on which efforts and strategies should be focused to prevent psychoactive substance use. By using various types of psychoactive substances, young people risk their health and safety. Perhaps users of such substances believe that this will help solve or minimise their problems. Such thinking leads to dangerous situations and further attempts to solve difficult issues using psychoactive substances will not only generate health problems, but may eventually lead to dependence. This is another problem to focus on when working with children and adolescents in the field of addiction prevention to provide them with psychological support so that they can solve their problems without reaching for psychoactive substances [1,30,31,32,33,34].

Preventive actions should be carried out in a planned and conscious manner towards children and young people in various social environments, especially by educational institutions and the family. The aim of prevention is to eliminate or reduce social threats resulting from various educationally unfavourable situations that disturb socialisation.

Training for parents and guardians should also be an essential element of socialisation. However, it is important to provide comprehensive education on this issue to children and adolescents, taking into account their developmental specificity and the risks resulting from it, and to recognise potential risk factors, such as mental illnesses and the abuse of legal and illegal substances. For these reasons, it is necessary to educate the environment cooperating with patients: health care workers, teachers and psychologists.

This research is limited by only three years of observation and the number of people in the study group. There were also no hospitalisations in the 5–8 age group. Another limitation of this study is the use of records only from medical records. Furthermore, this study used three scales to assess pain, related to the age of the patients—as a result, this matter can be distorted.

## 5. Conclusions

This three-year retrospective observational study has shown that girls are more likely to use stimulants such as alcohol and cigarettes, while boys are more likely to opt for designer drugs. The number of patients hospitalised more than once is increasing every year. Poisoning with psychoactive substances most often occurred by ingestion. The most severe intoxication was observed after alcohol consumption, while the most severe pain was reported by patients poisoned with other psychoactive substances.

## Figures and Tables

**Table 1:** Characteristics of the study group.

Gender Characteristics in the Study Group for Particular Years								
Gender	2016		2017		2018		Total	
	*n*	%	*n*	%	*n*	%	*n*	%
Male	40	42.11%	54	51.43%	51	49.51%	145	47.85%
Female	55	57.89%	51	48.57%	52	50.49%	158	52.15%
Total	95	100%	105	100%	103	100%	303	100%
The size of individual age groups and the numerical share of gender in the groups								
Age	Male			Female			Total	
	*n*		%	*n*	%		*n*	%
0–4 years	6		4.14%	3	1.90%		9	2.97%
9–10 years	2		1.38%	1	0.63%		3	0.99%
11–12 years	4		2.76%	11	6.96%		15	4.95%
13–14 years	30		20.69%	53	33.54%		83	27.39%
15–16 years	56		38.62%	55	34.81%		111	36.63%
17–18 years	47		32.41%	35	22.15%		82	27.06%
Total	145		100%	158	100%		303	100%
Place of residence								
Place of residence				*n*			%	
City				234			77.23%	
Village				69			22.77%	
Total				303			100%	
Place of education								
Place of education				*n*			%	
Kindergarten				2			0.66%	
School				279			92.08%	
School and educational centre				12			3.96%	
Does not attend school				10			3.30%	
Total				303			100%	

**Table 2:** Body temperature by blood alcohol levels.

Alcohol Level (‰)	Subnormal		Normal		Low-Grade Fever		Total	
	*n*	%	*n*	%	*n*	%	*n*	%
<1	6	13.04%	30	30.61%	5	38.46%	41	26.11%
1–1.5	6	13.04%	21	21.43%	5	38.46%	32	20.38%
>1.5–2	9	19.57%	20	20.41%	0	0.00%	29	18.47%
>2–2.5	15	32.61%	17	17.35%	2	15.38%	34	21.66%
>2.5–3	10	21.74%	5	5.10%	1	7.69%	16	10.19%
>3	0	0.00%	5	5.10%	0	0.00%	5	3.18%
Total	46	100%	98	100%	13	100%	157	100%
χ^2^ = 28.94, *p* < 0.001								

**Table 3:** Heart rate in the group of intoxicated patients by gender.

HR	Male		Female		Total	
	*n*	%	*n*	%	*n*	%
Normal	55	37.93%	43	27.22%	98	32.34%
Increased	85	58.62%	113	71.52%	198	65.35%
Decreased	5	3.45%	2	1.27%	7	2.31%
Total	145	100%	158	100%	303	100%
χ^2^ = 6.21, *p* < 0.04						

**Table 4:** Use of stimulants in the group of patients with symptoms of psychoactive substance intoxication by gender.

Stimulant *	Male		Female		Total		χ^2^	*p*
	*n*	%	*n*	%	*n*	%		
Alcohol	38	44.19%	48	55.81%	86	28.38%	0.64	0.42
Cigarettes	34	40.96%	49	59.04%	83	27.39%	2.18	0.13
Drugs	15	55.56%	12	44.44%	27	8.91%	0.70	0.40
Missing data	31	50.00%	31	50.00%	62	20.46%	0.14	0.70
Designer drugs	16	66.67%	8	33.33%	24	7.92%	3.74	0.053
No use	48	51.06%	46	48.94%	94	31.02%	0.56	0.45

* Children and adolescents ingest more than one substance.

**Table 5:** Use of stimulants in the group of patients with symptoms of psychoactive substance intoxication by age *.

Stimulant **	0–4 Years		9–10 Years		11–12 Years		13–14 Years		15–16 Years		17–18 Years		Total		χ^2^	*p*
	*n*	%	*n*	%	*n*	%	*n*	%	*n*	%	*n*	%	*n*	%		
Alcohol	0	0%	0	0%	3	3.49%	27	31.40%	30	34.88%	26	30.23%	86	28.38%	9.78	0.08
Cigarettes	0	0%	1	1.2%	4	4.82%	23	27.71%	26	31.33%	29	34.94%	83	27.39%	9.21	0.10
Drugs	0	0%	0	0%	0	0%	7	25.93%	10	37.04%	10	37.04%	27	8.91%	6.05	0.30
Missing data	0	0%	0	0%	3	4.84%	17	27.42%	29	46.77%	13	20.97%	62	20.46%	8.69	0.12
Designer drugs	0	0%	0	0%	0	0%	2	8.33%	14	58.33%	8	33.33%	24	7.92%	12.34	0.03
No use	9	9.57%	2	2.13%	7	7.45%	24	25.53%	31	32.98%	21	22.34%	94	31.02%	26.11	0.001

* There were no hospitalisations of patients in the 5–8 age group; ** Children and adolescents ingest more than one substance.

**Table 6:** The route of psychoactive substance intoxication in 2016–2018.

Route	2016		2017		2018		Total	
	*n*	%	*n*	%	*n*	%	*n*	%
Oral	82	86.32%	86	81.90%	85	82.52%	253	83.50%
Inhalation	13	13.68%	18	17.14%	17	16.50%	48	15.84%
Intravenous	0	0.00%	1	0.95%	0	0.00%	1	0.33%
Oral + Inhalation	0	0.00%	0	0.00%	1	0.97%	1	0.33%
Total	95	100%	105	100%	103	100%	303	100%
χ^2^ = 4.84, *p* < 0.56								

**Table 7:** Poisoning symptoms in the study group in 2016–2018.

Poisoning Symptoms	2016 *n* = 95		2017 *n* = 105		2018 *n* = 103		Total *n* = 303		χ^2^	*p*
	*n*	%	*n*	%	*n*	%	*n*	%		
Vomiting and nausea	43	45.26%	44	41.90%	37	35.92%	124	40.92%	1.85	0.39
Impaired consciousness	53	55.79%	46	43.81%	66	64.08%	165	54.46%	8.75	0.01
Behavioural disorders	43	45.26%	39	37.14%	46	44.66%	128	42.24%	1.73	0.42
Speech disorders	34	35.79%	38	36.19%	26	25.24%	98	32.34%	3.68	0.15
Impaired vision	0	0.00%	3	2.86%	6	5.83%	9	2.97%	8.02	0.02
Loss of consciousness	2	2.11%	34	32.38%	23	22.33%	59	19.47%	37.71	0.000001
Drowsiness	38	40.00%	26	24.76%	25	24.27%	89	29.37%	7.33	0.03
Dizziness	21	22.11%	27	25.71%	24	23.30%	72	23.76%	0.37	0.82
Loss of logical-verbal contact	22	23.16%	30	28.57%	24	23.30%	76	25.08%	1.02	0.59
Convulsions	4	4.21%	2	1.90%	3	2.91%	9	2.97%	0.92	0.63
Abdominal pain	8	8.42%	12	11.43%	6	5.83%	26	8.58%	2.1	0.34
Aggressive behaviour	13	13.68%	11	10.48%	26	25.24%	50	16.50%	8.75	0.02
Hypothermia	26	27.37%	22	20.95%	6	5.83%	54	17.82%	18.95	0.00008
Tremor	5	5.26%	7	6.67%	13	12.62%	25	8.25%	3.9	0.14
Gait disturbances	25	26.32%	50	47.62%	28	27.18%	103	33.99%	13.08	0.002
Total	95	100%	105	100%	103	100%	303	100%		

**Table 8:** An analysis of consciousness/mental state at the time of admission in the group of patients with symptoms of psychoactive substance poisoning in 2016–2018.

Consciousness/Mental State on Admission	2016		2017		2018		Total	
	*n*	%	*n*	%	*n*	%	*n*	%
Conscious	75	78.95%	79	75.24%	84	81.55%	238	78.55%
Unconscious	18	18.95%	20	19.05%	17	16.50%	55	18.15%
Other (intubated)	2	2.11%	6	5.71%	2	1.94%	10	3.30%
Total	95	100%	105	100%	103	100%	303	100%
χ^2^ = 3.17, *p* < 0.52								

**Table 9:** Comparison of GCS scores by gender and type of poisoning using the Kruskal–Wallis H test.

GCS					
	*n*	$\bar{x}$	Me	H	*p*
Gender					
Male	145	1.21	1	0.14	0.7
Female	158	1.22	1		
Type of poisoning					
Alcohol	131	1.30	1	9.1	0.01
Psychoactive substances	146	1.15	1		
Mixed	26	1.15	1		

**Table 10:** The severity of poisoning by gender and poisoning type (Kruskal–Wallis H test).

Severity					
	*n*	$\bar{x}$	Me	H	*p*
Gender					
Male	145	2.01	2	0.17	0.67
Female	158	1.97	2		
Type of poisoning					
Alcohol	131	2.12	2	12.2	0.002
Psychoactive substances	146	1.87	2		
Mixed	26	2.00	2		

**Table 11:** Pain on admission by gender and type of poisoning (Kruskal–Wallis H test).

Pain Severity on Admission					
	*n*	$\bar{x}$	Me	H	*p*
Gender					
Male	145	0.28	0	0.08	0.77
Female	158	0.36	0		
Type of poisoning					
Alcohol	131	0.18	1	8.87	0.01
Psychoactive substances	146	0.45	1		
Mixed	26	0.35	1		

## Data Availability

Data will be made available upon request to the corresponding author.
